# Rat in vitro spermatogenesis promoted by chemical supplementations and oxygen-tension control

**DOI:** 10.1038/s41598-021-82792-2

**Published:** 2021-02-10

**Authors:** Takafumi Matsumura, Takuya Sato, Takeru Abe, Hiroyuki Sanjo, Kumiko Katagiri, Hiroshi Kimura, Teruo Fujii, Hiromitsu Tanaka, Masumi Hirabayashi, Takehiko Ogawa

**Affiliations:** 1 Laboratory of Biopharmaceutical and Regenerative Sciences, Institute of Molecular Medicine and Life Science, Yokohama City University Association of Medical Science, Yokohama, Kanagawa Japan; 2 Department of Urology, Yokohama City University School of Medicine, Yokohama, Kanagawa Japan; 3 Department of Mechanical Engineering, Tokai University, Hiratsuka, Kanagawa Japan; 4 Institute of Industrial Science, University of Tokyo, Bunkyo, Tokyo Japan; 5 Faculty of Pharmaceutical Sciences, Nagasaki International University, Sasebo, Nagasaki Japan; 6 Center for Genetic Analysis of Behavior, National Institute for Physiological Sciences, Okazaki, Aichi Japan

**Keywords:** Spermatogenesis, Developmental biology

## Abstract

In vitro spermatogenesis (IVS) using air–liquid interphase organ culture method is possible with mouse testis tissues. The same method, however, has been hardly applicable to animals other than mice, only producing no or limited progression of spermatogenesis. In the present study, we challenged IVS of rats with modifications of culture medium, by supplementing chemical substances, including hormones, antioxidants, and lysophospholipids. In addition, reducing oxygen tension by placing tissues in an incubator of lower oxygen concentration and/or applying silicone cover ceiling on top of the tissue were effective for improving the spermatogenic efficiency. Through these modifications of the culture condition, rat spermatogenesis up to round spermatids was maintained over 70 days in the cultured tissue. Present results demonstrated a significant progress in rat IVS, revealing conditions commonly favorable for mice and rats as well as finding rat-specific optimizations. This is an important step towards successful IVS in many animal species, including humans.

## Introduction

Spermatogenesis is a complex cell differentiation process comprising an expansive cell proliferation, chromosomal recombination, reductive cell division to produce haploid cells, dynamic changes in cellular morphology and nuclear chromatin contents, and shedding off of residual body. These events happen sequentially taking quite long periods of time, more than a month in most mammals^1^. This process is naturally thought to have been tuned in each species to yield maximum reproductive outcome during the evolutional time span. The mechanism of spermatogenesis is still largely unknown and has yet to be elucidated in detail. This ignorance of ours becomes obvious when we try to reproduce the spermatogenesis in an artificial condition out of the body. In fact, spermatogenesis in most animal species cannot be reproduced *in vitro*^2^. However, IVS should become possible, if we had information of its mechanistic details, especially nutritional demands, signal cues, and optimal conditions regarding physical parameters. Conversely, if IVS becomes feasible and widely applicable, it could provide an experimental system for detailed analysis of spermatogenesis, which contributes to a more complete and comprehensive understanding of spermatogenesis. It would also provide applications to clinical problems in male infertility. In particular, fertility preservation in male pediatric cancer patients becomes feasible, if sperm production from cryopreserved testicular tissues becomes possible. Thus, developing systems for IVS is now a prime theme in the reproductive biology and related areas.

In 2011, we for the first time succeeded in producing mouse sperm from spermatogonial stem cells using an organ culture method^3^. The key to this achievement was a serum replacement, named Knockout Serum Replacement (KSR), supplemented in the culture medium. Soon later, the critical ingredients in KSR was discovered to be AlbuMAX, a purified serum albumin^3^. Because of this simple trick, the IVS with mouse testis tissue culture was repeated by several independent research groups and now becoming a standard procedure^4–8^. We continued working on the IVS to improve its efficiency by, for one thing, introducing microfluidic system. It was shown possible to induce and maintain spermatogenesis for more than 6 months^9,10^. However, all these progresses were restricted to experiments using mouse testis. Experiments using animals other than mice, including human, have been limited and mostly unsuccessful^11–16^. In case of rats, to our best knowledge, three reports dealt with IVS using organ culture method recently^17–19^. Reda A. et al*.* reported the formation of round spermatids in cultured rat tissue^17^. Using almost the same method, however, we and other groups hardly observed haploid cell formation, only found spermatocytes, meiotic germ cells, as the most differentiated germ cells in the cultured rat testis tissues^19,20^. It appeared that culture medium, namely αMEM plus KSR or AlbuMAX, which worked for mouse spermatogenesis, had limited or unstable effect on rat spermatogenesis. We needed definitely to improve or optimize culture medium for rat spermatogenesis. However, improving the culture medium was difficult, because the information on AlbuMAX, about its ingredients, was limited. The content of AlbuMAX is 97% albumin, but it contains myriad of substances derived from bovine serum, in which critical factors for IVS were hiding. We therefore examined AlbuMAX, using omics technologies, in comparison to bovine serums and bovine serum albumins of other sources. Then, we found that antioxidative substances, α-tocopherol, ascorbic acid, and glutathione, and lysophospholipids, particularly lysophosphatidylcholine (LPC), lysophosphatidic acid (LPA), and lysophosphatidylserine (LysoPS), were critically effective for the induction and promotion of mouse spermatogenesis. By supplementing these substances in the culture medium, mouse spermatogenesis was induced in neonatal mouse testis even without using AlbuMAX^21,22^.

In the present study, we challenged rat IVS based on these recent advancements in mouse experiments. The rat is one of the most commonly used experimental animals for wide range of biological and medical disciplines, particularly in the area of endocrinology, neurology, toxicology, and reproductive biology^23^. In researches on spermatogenesis, rat has been used most favorably as a model animal. Toxicological examination on testis has been dominated by experiments using rats. Thus, the chemical testing guide line issued by the organization for economic co-operation and development (OECD) recommends rats as most appropriate for animal experiments^24^.

On commencing the research on rat IVS, we have made a line of transgenic rat harboring fluorescent Venus gene, which is expressed in meiotic germ cells. With this transgenic rat, evaluation of spermatogenesis became reliable and concise. We searched for optimal culture conditions and succeeded in producing haploid round spermatids faithfully in vitro.

## Result

### Production of *Haspin*-Venus Transgenic (HV-Tg) rats

**Figure 1:**
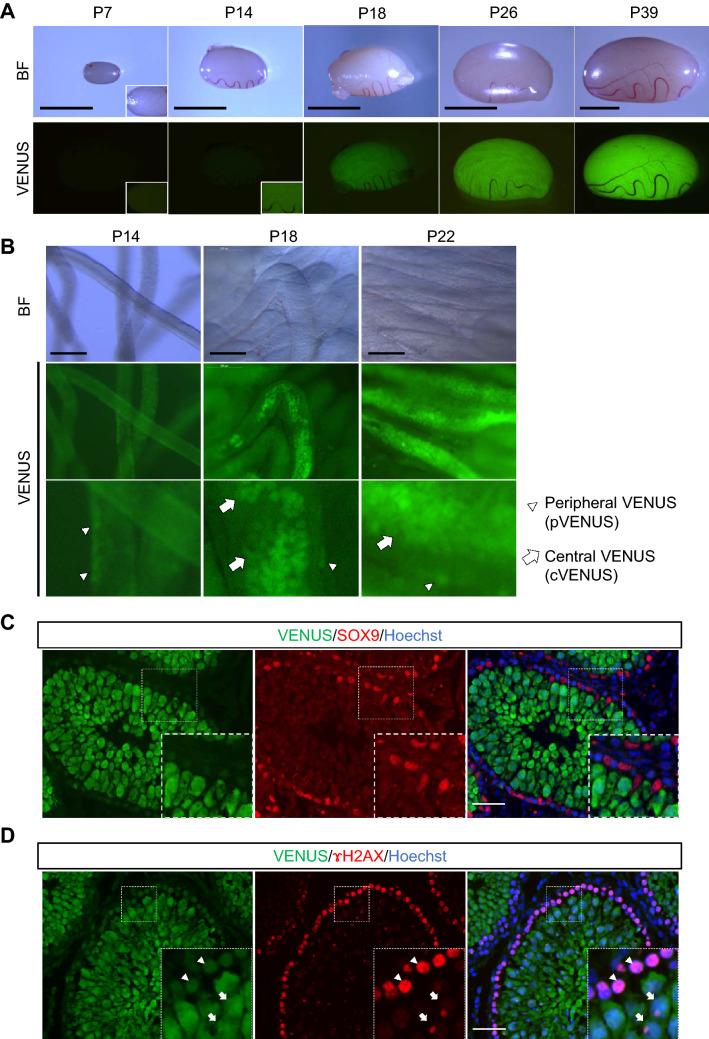
Characterization of *Haspin*-Venus Tg rats. (**A**) Testis of HV-Tg rats at age P7 to P39. Blight field pictures and Venus fluorescence under excitation light are shown. Scale bar; 5 mm. (**B**) Higher magnified view of HV-Tg rat testis. White arrowheads and arrows indicate pVenus and cVenus, respectivly. Scale bar; 0.2 mm. (**C**) Immunohistochemistry to Venus and SOX9, a marker of Sertoli cells, with Hoechst nuclear stain was performed on testis of rat P35. Venus stain was specific to germ cells, not appeared in Sertoli cells. Scale bar; 50 µm. (**D**) Immunohistochemistry to Venus and γH2AX with Hoechst stain was shown. White arrowheads indicate leptotene/zygotene spermatocytes with weaker Venus expression. White arrows indicate pachytene spermatocytes with stronger Venus expression. Scale bar; 50 µm.

In order to monitor the progression of rat spermatogenesis faithfully and concisely, we have produced a line of transgenic rats whose spermatogenic cells express Venus at its particular stage. We constructed a cassette of *Haspin*-promoter sequence flanked to Venus gene (Supplementary Fig. 1) and injected it into fertilized rat oocytes. The testis of this transgenic line, HV-Tg rat, expressed weak Venus around 2-week-old, and stronger Venus at around 3-week-old (Fig. 1A). On closer observation, the weak Venus expression was lining the edge of seminiferous tubule and observed since around P14. Then after around P18 onward, the stronger Venus scattered in the central region of the seminiferous tubule appeared, which became connected to be linear and wide throughout the whole testis in several days (Fig. 1A,B). We named these Venus expressions peripheral Venus (pVenus) and central Venus (cVenus), respectively. We examined these Venus expressions with immunohistochemistry and confirmed that they were confined to germ cells, and not observed in Sertoli cells (Fig. 1C). When comparing with the expression of γH2AX, whose staining showed broad nuclear pattern at leptotene to zygotene spermatocyte, followed by aggregation into the XY body in pachytene spermatocyte^25–27^, the weak Venus expression corresponded to leptotene to zygotene stage, whereas the strong expression matched to pachytene stage (Fig. 1D). From these findings, we concluded that pVenus and cVenus observed under stereomicroscope correspond to leptotene/zygotene spermatocytes and pachytene spermatocytes onwards, respectively.

### Modification of culture conditions for rat IVS

**Figure 2:**
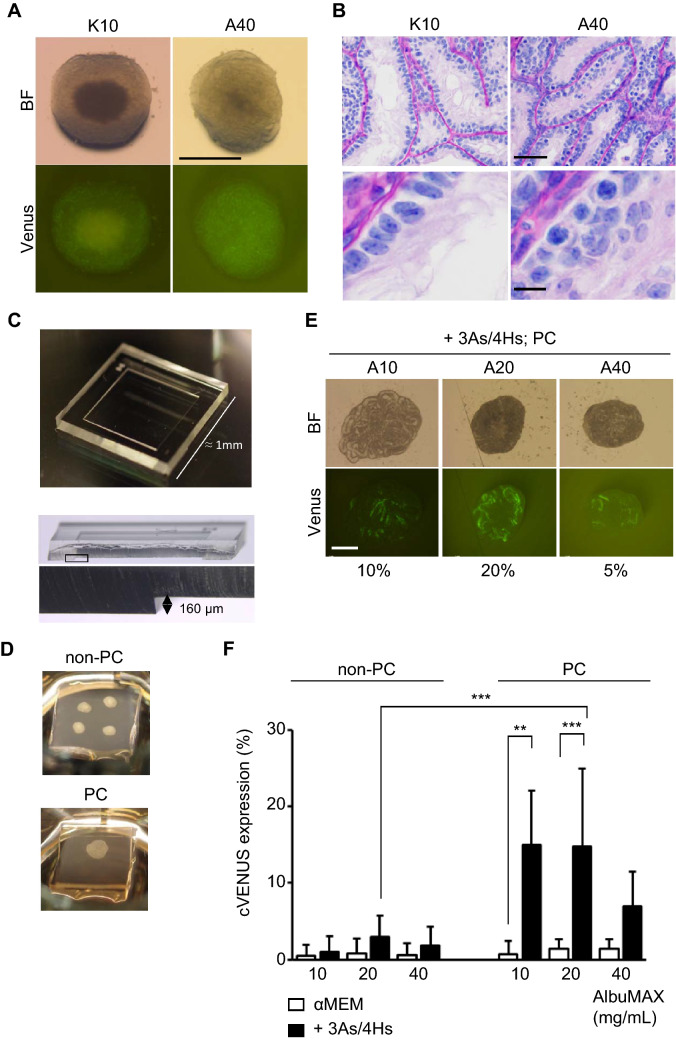
Three modification of culture conditions that promoted cVenus expression*.* (**A**) HV-Tg rat testis tissues cultured with αMEM plus 10% KSR (K10) or AlbuMAX 40 mg/mL (A40) at week 6. Scale bar; 1 mm. (**B**) Histological examination of cultured rat testis tissue at week 6. Scale bar; 50 µm (top panel), 10 µm (bottom panel). (**C**) Photo of a PDMS ceiling chip, having the 160 µm depth of dent in the center. (**D**) Pictures of organ culture set-up of non-PC and PC methods. Four tissue fragments were on the agarose gel in this case of non-PC method. With the PC method, a single tissue fragment was flattened between agarose gel and PC. (**E**) Photos of HV-Tg rat testis tissues cultured for 6 weeks with media containing 3As, 4Hs and different concentrations of AlbuMAX, with PC application. cVenus expression area ratios were shown under each picture. Scale bar; 1 mm. (**F**) Average cVenus expression area ratio in each group was shown in bar graph with standard deviation. Evaluated at 6 weeks of culture. n = 5–36 (P3–P9). Statistical significance; ***P < 0.001. **P  < 0.01.

We cultured tissue fragments of HV-Tg rat testes, obtained from P3–P9 pups, by the gas–liquid interphase method using an agarose gel stand^3^. The progression of spermatogenesis was monitored by the Venus expression every week. Initially, the standard culture medium used for mouse testis tissue, α-MEM supplemented with 10%KSR (K10) or AlbuMAX at 40 mg/mL (A40), was tested. These media, however, hardly induced cVenus expression in rat testis tissues (Fig. 2A). Histological examination showed several spermatocytes, leptotene to zygotene stages, in a few seminiferous tubules. The majority of the tubules, however, contained only a few spermatogonia and rest are composed of only Sertoli cells (Fig. 2B). Namely, the culture medium which worked efficiently to induce mouse spermatogenesis turned out ineffective for rat spermatogenesis. Then, we modified the culture condition in the following three points. Firstly, we added antioxidants of α-tocopherol (αT), Ascorbic acid (AA), and glutathione (GSH), in the culture medium, the combination of which is hereafter referred to as 3 antioxidants (3As). We also added hormones, consisting of testosterone (T), triiodothyronine (T_3_), luteinizing hormone (LH), and follicle stimulating hormone (FSH), which was named 4 hormones (4Hs). These supplements had been shown to be effective in promoting mouse spermatogenesis^21,22^. Secondly, we applied silicone cover ceiling method, which used polydimethylsiloxane (PDMS) ceiling (PC) chip having 160 µm-depth square dent, to hold the testis tissue in the dent space making it thin and flat on the agarose gel (Fig. 2C,D)^28^. Thirdly, we tested different concentrations of AlbuMAX from 10 to 40 mg/mL. Experiments examined these modifications showed that these three all had significant effect on the induction of cVenus (Fig. 2F, Supplementary Table 3A). In particular, supplementing 3As/4Hs and covering with PC chip was significantly effective in increasing cVenus expressing area in each tissue (Fig. 2E). As for the concentration of AlbuMAX, 40 mg/mL which was regularly used for mice as optimal turned out to be a bit higher than optimal for rats and lower concentration of 20 mg/mL appeared to be preferable.

### Cooperative effect of antioxidants and hormones

**Figure 3:**
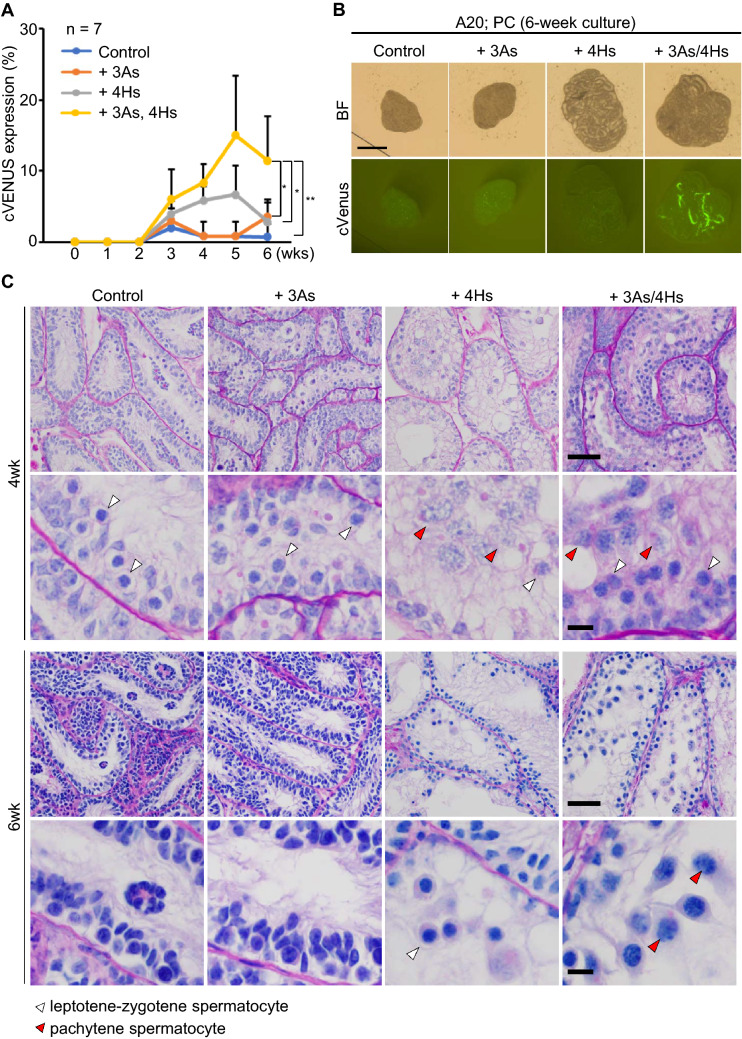
Antioxidants and hormones cooperatively promoted in vitro rat spermatogenesis. (**A**) The time course of cVenus expression area ratio of 4 groups was shown in a line graph. The culture medium of control group was αMEM + AlbuMAX (20 mg/mL). n = 7 (P4 and P7). Statistical significance; **P  < 0.01. *P  < 0.05. (**B**) Photos of cultured tissues at week 6. Scale bar; 1 mm. (**C**) PAS staining histological pictures of cultured tissues at 4 and 6 weeks. White and red arrowheads indicate leptotene/zygotene and pachytene spermatocytes, respectively. Magnified images are shown in 2nd and bottom row. Scale bar; 50 µm (top and 3rd row). Scale bar; 10 µm (2nd and bottom row).

To study the individual effects of 3As and 4Hs, we added each of them separately and monitored the cVenus expression for 6 weeks. The addition of 3As showed little effect on the induction of cVenus expression, whose cVenus expressing area was similar to that in control group. The 4Hs showed a minor effect on the induction of cVenus expression (Fig. 3A, Supplementary Table 3B). Interestingly, combination of 3As and 4Hs increased the cVenus expression significantly (Fig. 3A,B). Histological examination at week 4 showed that all groups contained leptotene/zygotene spermatocytes, but pachytene spermatocytes were only observed in groups that medium was supplemented with 4Hs and 3As/4Hs. This suggested that 4Hs promoted meiotic progression, while 3As did not. However, by week 6, number of meiotic germ cells in 4Hs-added group appeared to have decreased, while in 3As/4Hs-added group such germ cell loss appeared minor or milder (Fig. 3C). This finding suggested that 3As functioned as expected to protect germ cells from oxidative stress. Thus, it was concluded that the combination of 3As and 4Hs, each having different effect on germ cells, enhanced cVenus expression.

### Effect of PDMS ceiling (PC) and oxygen concentration

**Figure 4:**
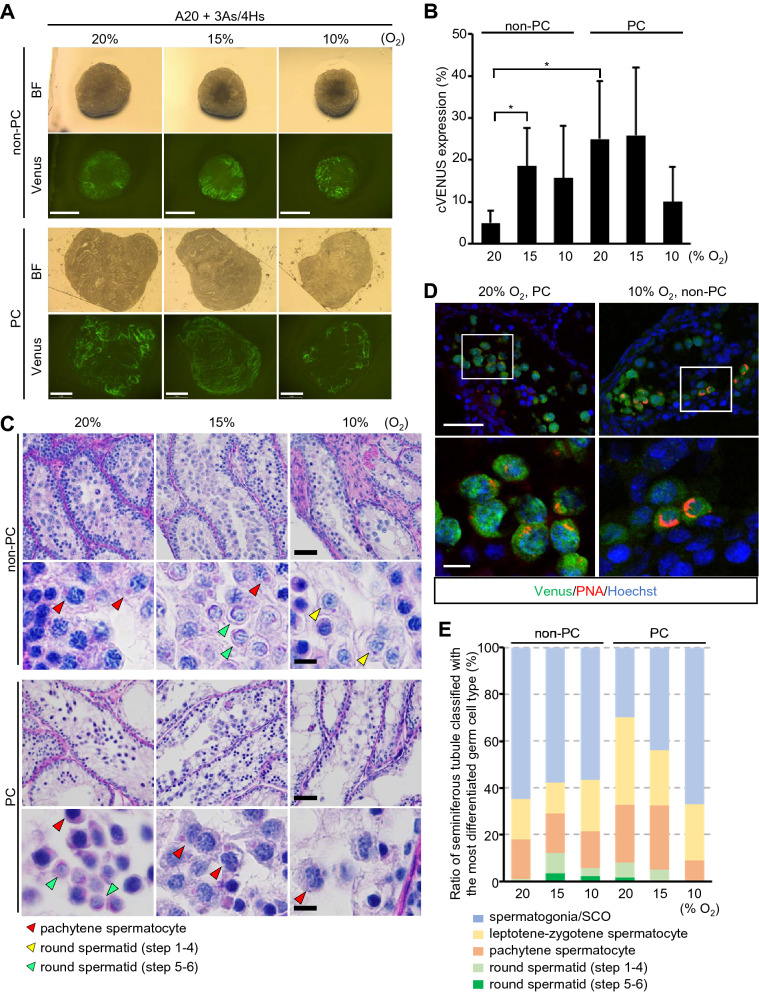
Effect of oxygen concentration for in vitro rat spermatogenesis. (**A**) Photos of cultured testis tissues at week 6 in different O_2_ concentrations with or without PC. Scale bar; 1 mm. (**B**) cVenus expression area ratio in each group at week 6. n = 6–7 (P7). *P  < 0.05. (**C**) Histological pictures of 6 week cultured tissue stained with PAS. Red, yellow, and green arrowheads indicate pachytene spermatocyte, round spermatids at step 1–4, and step 5–6, respectively. Panels in 2nd and bottom row are magnified image. Scale bar; 50 µm (top and 3rd row), 10 µm (2nd and bottom row). (**D**) Immunohistochemical staining with anti-Venus (green) and PNA (red), along with nuclear stain with Hoechst (blue). Area in white rectangles in top panels was enlarged in bottom panels. Scale bar; 50 µm (top panels), 10 µm (bottom panels). (**E**) Ratio of seminiferous tubules classified with the most differentiated germ cell type contained was shown by band graph. Round spermatids of step 1–4 appears as having acrosome of dot shape, while step 5–6 having cap shape. n = 5–12 (P7).

Next, we evaluated the effect of PC in more detail. As PC spreads the tissue thin and flat on agarose gel, it would be reasonable that every part of a tissue can enjoy smooth and equal supply of nutrients and oxygen. This simple modification of the tissue form in relation to the access of nutrients and oxygen could have contributed to the increased cVenus expression area and promotion of spermatogenesis. In addition, even though PDMS claimed high oxygen permeability, it was estimated that PC interfered with the oxygen supply to the tissue and slightly reduced the oxygen tension in and around the cultured tissue. We reasoned that this sort of a hypoxic condition could be rather beneficial for spermatogenesis, because the 20% O_2_ in the incubator is much higher than physiological in vivo condition. Then, we tested different concentration of O_2_ in the incubator with and without PC. First, without PC, we found that 15% O_2_ increased cVenus expression area significantly compared to 20%. With 10% O_2_, the cVenus expression area was also higher than with 20% O_2_ (Fig. 4A,B, Supplementary Table 3C). This seemed indicated that 20% O_2_ is not optimal and too high for testis tissues culturing for spermatogenesis. On the other hand, in PC group, cVenus expression areas under O_2_ concentration 15% and 20% were comparable, while in 10% O_2_ the expression area of cVenus decreased. These results can be interpreted that when the PC chip is placed on top of the tissue, the oxygen concentration around the tissue is somewhat lower than outside the PC. Therefore, when the tissue is placed in a 10% O_2_ incubator and covered with a PC chip, the tissue will be exposed to less than 10% oxygen concentration, which seemed too hypoxic for the tissue. Histological examination with PAS staining revealed pachytene meiotic spermatocytes in all non-PC and PC groups. Haploid round spermatids were mainly observed in 15% and 10% O_2_ non-PC groups and 20% O_2_ PC group (Fig. 4C). When spermatids were sub-classified into step 1–4 and 5–6, based on the shape of acrosome, each having dots and cap-like forms, respectively, spermatids in step 5–6 were most frequently observed in 10 and 15% O_2_ non-PC groups, with frequency of over 50% (Supplementary Table 2). The presence of round spermatids was also confirmed by immunohistochemical analysis using lectin PNA, which react with acrosome (Fig. 4D). The progression of spermatogenesis was quantitatively analyzed by the presence of spermatogonia, spermatocytes, and spermatids in each seminiferous tubule. This validated the data of cVenus expression area and demonstrated that non-PC in 15% O_2_ and PC in 20% O_2_ supported the progression of spermatogenesis most efficiently (Fig. 4E, Supplementary Table 3D).

### Effect of lysophospholipids

**Figure 5:**
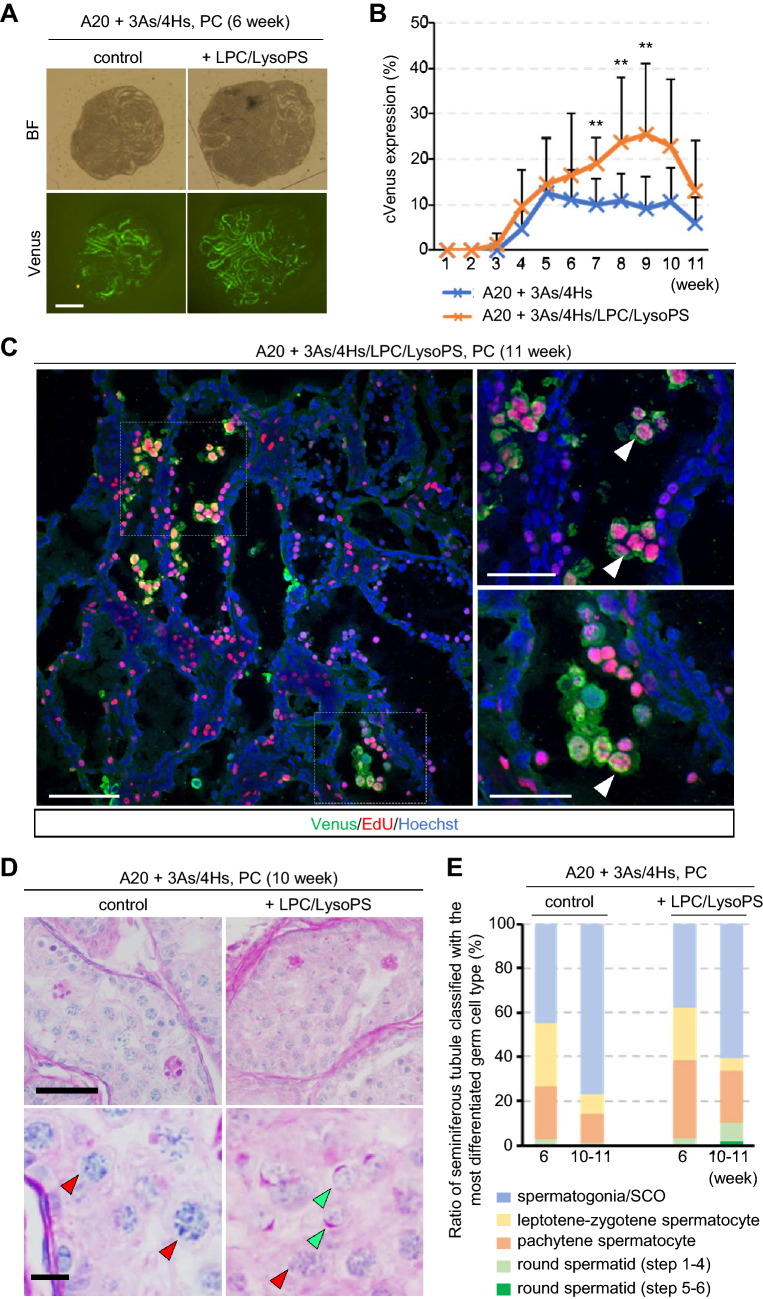
Effect of lysophospholipids in the longer-term IVS. (**A**) Tissues cultured with medium containing AlbuMAX (20 mg/mL) + 3As/4Hs, and with or without LPC/LysoPS, pictured at week 6. Scale bar; 1 mm. (**B**) Time course of cVenus expressing area ratio for 11 weeks. n = 12 (P7 and P9). Statistical significance; **P  < 0.01. (**C**) EdU was added in the medium for 1 day at week 8, fixed and stained at week 11, followed by immunohistochemical staining with Venus, EdU, and Hoechst. White arrowheads indicate cells positive for both Venus and EdU. Dashed line rectangle areas in the left panel were enlarged in the right panels. Scale bar; 100 µm (left), 50 µm (right). (**D**) PAS stained histological view of cultured tissues, with or without LPC/LysoPS, at week 11. Red and green arrowheads indicate pachytene spermatocytes and step 5–6 round spermatids, respectively. Bottom panels are magnified image of top panels. Scale bar; 50 µm (top panels), 10 µm (bottom panels). (**E**) Ratio of seminiferous tubules classified with the most differentiated germ cell type. n = 5–8 (P4–P9).

In our previous study, lysophosoplipids was shown to be effective in inducing/promoting mouse IVS^22^. So, we investigated if the lysophopholipids are effective to rat IVS as well. In the present study, LPC and LysoPS were supplemented in the culture medium, which consisted of αMEM, AlbuMAX (20 mg/mL), 3As and 4Hs. In 6 weeks of culture, the medium added with LPC/LysoPS induced cVenus expression a bit higher than the medium without them (Fig. 5A). Then, the effect of LPC/LysoPS became evident with prolonged culture period as long as 11 weeks (Fig. 5B, Supplementary Table 3E). This long duration suggested that the IVS represented not only the so-called first wave of spermatogenesis but also a continuous cycle of spermatogenesis as well. To confirm this, EdU was added in the medium at week 8 and tissues were harvested at week 11. The samples were immunohistochemically stained with anti-EdU and anti-Venus antibodies, which showed double positive cells in the center of seminiferous tubules (Fig. 5C). This indicated that preleptotene spermatocyte or spermatogonia at week 8 developed into pachytene or later stages of spermatogenic cells at week 11, suggesting not only first wave spermatogenesis but also subsequent cycles took place. Histological examination with PAS stain at week 10 also demonstrated meiotic spermatocytes and round spermatids (Fig. 5D). Although seminiferous tubules containing round spermatids were not many, they were observed in both week 6 and 10–11 (Fig. 5E, Supplementary Table 3F), with frequency as high as 80% (Supplementary Table 2).

### IVS with Sprague–Dawley strain rats

It was reported recently that mouse strain difference drastically influenced the efficiency of IVS with organ culture method^6^. It would be possible therefore that the result of rat IVS differs in different strains. As HV-Tg rat was Wistar strain, we performed additional experiments using Sprague–Dawley (SD) strain. We cultured wild-type SD rat testicular tissues with the medium of αMEM supplemented with K10 and A20 + 3As/4Hs/LPC/LysoPS. With K10 medium, we found a few preleptotene or zygotene spermatocytes in some seminiferous tubules, but no haploids were observed in cultured testis tissues under any conditions, including 20% or 15% O_2_ and PC or non-PC, in 6 week-culture (Supplementary Fig. 3A,B). On the other hand, with the medium of A20 + 3As/4Hs/LPC/LysoPS, we found pachytene spermatocytes under every condition, and round spermatids under 15% O_2_/PC condition (Supplementary Fig. 3C,D, Supplementary Table 3G). It was shown therefore that our new culture condition effectively induced IVS of not only Wistar rats but also SD rats.

## Discussion

Rats have been the most common, popularly used experimental animal in various biomedical research areas, including reproductive biology^29^. Although mice might have replaced the position of rats as a representative rodent owing to their faster progress in genetics and the availability of genetically modified mouse lines, the importance of rats is yet recognized in many research areas^30^. In toxicology, rats continue to be main and important animal species for testing chemicals and drugs^24,31^. Body size of rats, ten times larger than mice, allows a variety of organ transplantation experiments more easily than mice, while they have advantages over dogs and pigs for instance in terms of cost and the availability of genetic modification technology^32^. As for spermatogenesis, rats have long been the standard experimental model animal^33^. In studies on IVS, rats along with rabbits and guinea pigs were used initially in history and rats remained to be the main experimental animal^34,35^. In 2011, we developed an organ culture method for spermatogenesis using mice, resulting in the production of fertile sperm from spermatogonial stem cells^3^. This study was initiated in repeating the studies of Steinberger’s^35,36^, and spermatogenic progression was surprisingly enhanced by supplementing the culture medium with KSR or AlbuMAX^3^. This simple secret of success, at that time, made us suppose that it should apply not only to mice but also to rats and many other species as well. However, it was not the case. Culture experiments using the same method with rat testis tissues showed only limited progression of spermatogenesis. Namely, effect of KSR/AlbuMAX was not appreciated in our experiments using rat testis, disappointing us deeply.

So, why is in vitro spermatogenesis so difficult in rats? The reason is not clear, but we can present similar experimental difficulties in working with rat cells and organs. For example, establishing rat ES cells was difficult and took another 30 years from the first reports of mouse ES cells^37–41^. Rat oocytes have been reported to be fragile and easily activated in vitro, making in vitro fertilization of rat by ICSI or ROSI very difficult. For the same reason, nuclear cloning of rats was difficult and only one case of rat cloning has been reported^42^. Although technical improvements, in such as ICSI and ROSI^43,44^, have eliminated many of these difficulties, in vitro experiments with rat samples remain a challenging task. Therefore, in vitro spermatogenesis would be just another example of the difficulty of experimenting with rats.

Our data has shown that some modifications of culture condition improve the efficiency of rat IVS significantly. The modification consisted of supplementing four hormones, three antioxidants, and two lysophospholipids to the culture medium. It also contained covering the tissue with PC chip and/or reducing oxygen concentration to 15% in the incubator. With these modifications, rat IVS proceeded up to the haploid cell formation, precisely the production of round spermatids at step 5–6. Furthermore, we observed round spermatids in testicular tissue cultured for 11 weeks, along with EdU-positive meiotic germ cells which took up the EdU at week 8 of culture. These results suggest that our IVS system supported spermatogenesis not only of so called first-wave but also from stem cells dividing in vitro.

The importance of vitamin E for rat spermatogenesis in vivo was reported that vitamin E deficient diet impaired spermatogenesis^45,46^. It was also reported by the Steinbergers that vitamins A, C, and E, as well as FSH and hCG supplemented in medium have advanced rat IVS^47^. These substances were effective in mouse IVS, too. We found in our previous studies that hormones, antioxidants, and lysophospholipids examined in this study had a definite effect on improving IVS in mice^21,22^. Thus, their effects are not specific to rats, but are common to mice as well.

On the other hand, some modifications were explicitly more effective in rats than in mice. In particular, the effect of PC and 15% O_2_ was clearly demonstrated in rats. It is generally recognized that oxygen concentration in regular culture condition (20%) is too high, considering that is much lower in vivo. In fact, in culture practice for fertilized oocytes or embryos, 5% O_2_ had been shown beneficial and has been generally applied^48,49^. In case of organ/tissue culture, however, situation is different. Tissue has a certain volume, and the oxygen tension (pO2) of each region of the tissue varies greatly. If the peripheral areas of the tissue enjoy adequate pO_2_, the inner areas will naturally suffer from oxygen deficiency. If certain inner regions are in adequate pO_2_, the outer regions will suffer from oxygen toxicity in the long run. Thus, oxygen is a very delicate issue in organ culture experiments. In this study, we applied PC chip to flatten the testis tissue into 160 µm thickness. This may have alleviated pO2 differences between locations within testicular tissue, allowing more uniform induction of spermatogenesis. There were also trials to culture isolated seminiferous tubules rather than tissue masses. In this case, each seminiferous tubule could be in nearly same pO_2_, being able to evaluate the pO_2_ effect on IVS more reliably^50^. By lowering O_2_ in the incubator, in the present study, we investigated the effects of O_2_ concentration and found that about 15% of O_2_ was optimal for inducing spermatogenesis in rats. However, in vivo testicular O_2_ is much lower than 15%. The testicular interstitial pO_2_ in rats was reported to be around 12.5 mmHg, or 1.6% O_2_ under atmospheric pressure^51^. Since rat IVS seems more sensitive to oxidative stress than mouse IVS, it would be worth refining antioxidant strategies for further improvement.

It is certainly important to study IVS with different animal species for comparison and to extract principles of spermatogenesis. Previous reports using different animal species, including cats, marmosets, and humans, examined variety of culture conditions^11,13–16^. In particular, effect of hormones has been extensively examined, with some reporting positive effects and others not^13–15,19^. These are precious data, but needs careful interpretation. In fact, a practical difficulty in culture experiment is that the experiment is ruined if only even a single essential factor is missing or deteriorated in the medium. Our present study using rat testis revealed such essential factors and appropriate conditions for rat IVS. Nonetheless, it is not possible yet to produce elongating spermatids and sperm. In addition, progeny production with ROSI remains to be tested to confirm the competency of those round spermatids to generate offspring. Establishing a protocol for more elaborated rat spermatogenesis in vitro would provide a valuable platform for spermatogenesis research as well as applications in reproductive toxicology.

## Materials and methods

### Production of transgenic rats

SD rats were purchased from Japan SLC. *Haspin*-Venus transgenic (HV-Tg) rats (Wistar strain) was produced as follows. The transgene construct was shown in Supplementary Fig. 1. The sequence of the rat *Haspin* bidirectional promoter was derived from NCBI Reference Sequence: NC_005109.3. It was injected into fertilized rat oocytes to obtain offspring after digestion by DraIII and AflII. Venus positive rats were selected for breeding. The primers below were used to detect the transgene. Forward primer; 5′–TACGGCAAGCTGACCCTGAA–3′. Reverse primer; 5′–TGTGATCGCGCTTCTCGTTG–3′. Rat were housed in air-conditioned rooms, 24 ± 1 °C and 55 ± 5%, with 14 h light and 10 h dark lightning cycle. Animals were kept in a specific pathogen free room. The MF hard pellets (Oriental Yeast) were fed ad libitum. Drinking water was acidified to pH 2.8–3.0 by HCl^52^. All animal experiments conformed to the Guide for the Care and Use of Laboratory Animals and were approved by the Institutional Committee of Laboratory Animal Experimentation (Animal Research Center of Yokohama City University, Yokohama, Japan).

### Culture method

The testes of HV-Tg rats, postnatal days 3–9 (P3–P9), were decapsulated and gently separated by forceps into 8–20 pieces per testis. Tissue fragments were then placed on blocks of agarose gel in the wells of a culture plate. The size of each tissue piece on agarose gel block was 1–3 mm in diameters and around 200 µm in thickness. To make the agarose gel block, agarose powder (Dojindo Molecular Technologies) was dissolved in water purified with Milli-Q (35–1251, Merck) at 1.5% (w/v) and autoclaved. During cooling, 33 mL of agarose solution was poured into 10 cm dishes to form a 5 mm thick gel. The gel was cut into approximately 10 mm square pieces, which were used as stands for testis tissue placement. Gels were submerged in culture medium in 12 well culture plates (#353043, Corning Incorporated) for more than 6 h, twice, with a medium change in between. After medium removal, 0.5 ml of new medium was added to each well to half-soak gels, upon which testis tissues were placed. Each gel stand was loaded with 3–4 tissues. In cases that PC method was applied, single tissue was placed on a gel, and PC chip having 160 μm depth of dent was covered over the tissue. The PC was prepared based on our previous papers^28,53^. Briefly, PDMS prepolymer and curing reagent (Silpot 184; Dow Corning) were mixed at a 10:1 weight ratio. The 10 ml of mixture was poured over the mold master, then placed in a vacuum chamber for degassing for an hour and moved to an oven to be heated at 72 °C for 3 h for curing. After cooling down, solidified PDMS was peeled off from the master. This PDMS disk was diced into individual chips, about 10 mm square and 1 mm thick, using a knife. The mold master was produced as previously reported using conventional photolithography and soft lithography techniques^53–55^. Briefly, the material of the master mold, a negative‐type photoresist (SU‐82,100 & 2075; MicroChem Co.) was poured on a 4‐in. wafer and spincoated over it to evenly achieve the target thicknesses of 160 μm over the wafer. After prebaking, ultraviolet light was administered through a photomask to create different size of square space for tissue placement, followed by postbaking. The baked mold master was developed by incubation in propylene glycol monomethylether acetate (GODO, Tokyo, Japan) for 20–30 min, followed by rinsing in isopropanol (code 32435‐70; Kanto Chemical, Kanto, Japan). This SU‐8 mold master can be used repeatedly for the replica molding of PC chips. The photomask was designed with CAD software (AutoCAD 2018, academic free license; Autodesk Inc., San Rafael, CA) and fabricated with a laser lithography system. Medium was changed once a week. The culture incubator was supplied with 5% carbon dioxide, either in air (20% O_2_), 15% O_2_, or 10% O_2_, as mentioned in the text and maintained at 34 °C. The protocol was previously described in detail^52,56^. For EdU incorporation assay, the culture medium was changed to medium containing the 5 μM of EdU and incubated for 1 day. The next day, the medium was changed to regular medium without EdU. Tissues were collected 3 weeks later for immunohistochemistry.

### Culture medium preparation

To prepare AlbuMAX medium, AlbuMAX I (11,020–021, Thermo Fisher Scientific) was dissolved in double-concentrated α-MEM (12000–022, Thermo Fisher Scientific) at half the volume of the final solution, stirred until complete dissolution, followed by the addition of factors including T (20808341, Wako, 10 mM stock sol. in ethanol), T3 (T6397, Merck, 2.0 μg/mL stock sol. in ethanol), LH (L5259, Merck, 50 μg/mL stock sol. in Milli-Q water), FSH (F4021, Merck, 50 μg/mL stock sol. in Milli-Q water), L-Ascorbic acid 2-glucoside (092,375, Matrix Scientific, 1 M stock sol. in Milli-Q water), αT acetate (47786, Merck, 1 M stock sol. in ethanol), L-Glutathione (G6013, Merck, 100 mg/mL stock sol in Milli-Q water), L-a-Lysophosphatidylcholine (LPC)(L4129, Merck, 50 mg/mL stock sol. in ethanol), and 18:1 Lysophosphatidylserine (LysoPS)(858143P, Merck, 2 mM stock sol. in ethanol) at the concentrations indicated in Supplementary Table 1. Then, 7% NaHCO3 solution was added (0.026 ml/L) to achieve a final concentration of 1.82 g/L (0.0182 g for 10 ml of medium). Antibiotic–Antimycotic (15240062, Thermo Fisher Scientific) was added at a 1/100 volume to achieve a final concentration of 100 IU/ml for penicillin, 100 μg/mL for streptomycin, and 250 ng/ml for amphotericin. After sterilization with Millipore filter having pore size of 0.22 µm, media were stored at 4 °C.

### Venus expression observation

Cultured tissues were observed once a week under a stereomicroscope equipped with an excitation light for Venus (LeicaM205 FA; Leica). The extent of cVenus expression in each tissue sample was assessed on a seven point scale of 0%, 5%, 10%, 20%, 30%, 40%, and 50% or more (rated as 50%), judging visually the ratio of the area of cVenus expression in the whole region of the each tissue fragment (Supplementary Fig. 2).

### Histological and immunohistochemical examinations

In histological examinations, specimens were fixed with Bouin’s fixative and embedded in paraffin. Section for each specimen was stained with hematoxylin and periodic acid Schiff (PAS). The stage of germ cells was evaluated by their morphology and staining pattern of acrosome and nucleus^33^. In immunofluorescence staining, tissues were fixed with 4% paraformaldehyde in PBS at 4 °C overnight. Tissues were then soaked in solutions of 10, 15, and 20% (w/v) sucrose in PBS for 1 h each in succession for cryoprotection. They were cryo-embedded in OCT compound (Sakura Finetek Japan) and cut into 7 μm thick sections. The cryosections were washed with 0.2% PBT (0.2% Triton X–100 in PBS) four times and then treated with Image-iT FX Signal Enhancer (Thermo Fisher Scientific) for 30 min. Incubation with primary antibodies in Can Get Signal immunostain Solution A (TOYOBO) was performed overnight at 4 °C, followed by rinsing four times with PBS, and then secondary antibodies diluted in Can Get Signal immunostain Solution A were applied for 1 h at room temperature. The sections were washed with PBS and nuclei were counterstained with Hoechst33342 dye. Finally, sections were mounted by Prolong Diamond Antifade Mountant (Thermo Fisher Scientific) before observation. Antibodies used as primary antibody were anti-GFP (1:1000, ab13970, Abcam), anti-ɤH2AX (1:500, ab81299, Abcam) and anti-SOX9 (1:200, KO608, Trans Genic Inc.). Lectin PNA From Arachis hypogaea (peanut), Alexa Fluor 568 Conjugate (1:1000, L32458, Thermo Fischer Scientific) was used to identify acrosome. Antibodies used for secondary antibody were Alexa Fluor 488-conjugated goat anti-chicken antibody (A–11039, 1:200, Thermo Fischer Scientific), Alexa Fluor 555-conjugated goat anti-mouse antibody (A–21424, 1:200, Thermo Fischer Scientific), and Alexa Fluor 555-conjugated goat anti-rabbit antibody (A–21428, 1:200, Thermo Fischer Scientific). EdU detection was performed with Click-iT EdU Cell Proliferation Kit for Imaging, Alexa Fluor 555 dye (C10338, Thermo Fisher Scientific) before Image-iT FX Signal Enhancer. Observation of immunostained samples were performed with confocal microscope (FV1000–MPE, Olympus).

### Statistical analysis

Data on cVenus expression area ratio were shown as mean ± SD in graphs. Non-parametric multiple comparison tests, the Kruskal–Wallis test, followed by the Steel–Dwass test, were performed to assess the significance of differences in Venus expression area ratio (Figs. 2F, 3A, 4B).

## Supplementary Information

Supplementary Information 1.

Supplementary Information 2.

## References

[1] Clermont Y (1972). Kinetics of spermatogenesis in mammals: seminiferous epithelium cycle and spermatogonial renewal. Physiol. Rev..

[2] Komeya M, Sato T, Ogawa T (2018). In vitro spermatogenesis: a century-long research journey, still half way around. Reprod. Med. Biol..

[3] Sato T (2011). In vitro production of functional sperm in cultured neonatal mouse testes. Nature.

[4] Arkoun B (2015). Retinol improves in vitro differentiation of pre-pubertal mouse spermatogonial stem cells into sperm during the first wave of spermatogenesis. PLoS ONE.

[5] Nakamura N (2017). Evaluation of culture time and media in an in vitro testis organ culture system. Birth Defects Res..

[6] Portela JMD (2019). Strains matter: success of murine in vitro spermatogenesis is dependent on genetic background. Dev. Biol..

[7] Dumont L (2016). Vitamin A prevents round spermatid nuclear damage and promotes the production of motile sperm during in vitro maturation of vitrified pre-pubertal mouse testicular tissue. Mol. Hum. Reprod..

[8] Isoler-Alcaraz J, Fernández-Pérez D, Larriba E, Del Mazo J (2017). Cellular and molecular characterization of gametogenic progression in ex vivo cultured prepuberal mouse testes. Reprod. Biol. Endocrinol..

[9] Komeya M (2016). Long-term ex vivo maintenance of testis tissues producing fertile sperm in a microfluidic device. Sci. Rep..

[10] Komeya M (2017). Pumpless microfluidic system driven by hydrostatic pressure induces and maintains mouse spermatogenesis in vitro. Sci. Rep..

[11] de Michele F (2017). Preserved seminiferous tubule integrity with spermatogonial survival and induction of Sertoli and Leydig cell maturation after long-term organotypic culture of prepubertal human testicular tissue. Hum. Reprod..

[12] de Michele F (2018). Haploid germ cells generated in organotypic culture of testicular tissue from prepubertal boys. Front. Physiol..

[13] Medrano JV (2018). Influence of temperature, serum, and gonadotropin supplementation in short- and long-term organotypic culture of human immature testicular tissue. Fertil.. Steril..

[14] Portela JMD (2019). Assessment of fresh and cryopreserved testicular tissues from (pre)pubertal boys during organ culture as a strategy for in vitro spermatogenesis. Hum. Reprod..

[15] Heckmann L (2020). The initial maturation status of marmoset testicular tissues has an impact on germ cell maintenance and somatic cell response in tissue fragment culture. Mol. Hum. Reprod..

[16] Silva AF (2018). Can we induce spermatogenesis in the domestic cat using an in vitro tissue culture approach?. PLoS ONE.

[17] Reda A, Hou M, Winton TR, Chapin RE, Soder O, Stukenborg JB (2016). In vitro differentiation of rat spermatogonia into round spermatids in tissue culture. Mol. Hum. Reprod..

[18] Liu F (2016). Effect of KnockOut serum replacement on germ cell development of immature testis tissue culture. Theriogenology.

[19] Saulnier J*, et al.* Improving freezing protocols and organotypic culture: a histological study on rat prepubertal testicular tissue. *Ann. Biomed. Eng. *1–16 (2020). 10.1007/s10439-020-02535-8 32440757

[20] Nakamura, N. & Sloper, D. T. Comparison of germ cell differentiation of rat testis fragments cultured in knockout serum replacement versus Albumax™ I. *Birth Defects Res*. 10.1002/bdr2.1859 (2020). 10.1002/bdr2.1859 33348473

[21] Sanjo H (2018). In vitro mouse spermatogenesis with an organ culture method in chemically defined medium. PLoS ONE.

[22] Sanjo H (2020). Antioxidant vitamins and lysophospholipids are critical for inducing mouse spermatogenesis under organ culture conditions. FASEB J. Off. Publ. Fed. Am. Soc. Exp. Biol...

[23] Iannaccone PM, Jacob HJ (2009). Rats!. . Dis. Model Mech..

[24] *Test No. 421: Reproduction/Developmental Toxicity Screening Test*. OECD Publishing (2016).

[25] Dai MS, Hall SJ, Vantangoli Policelli M, Boekelheide K, Spade DJ (2017). Spontaneous testicular atrophy occurs despite normal spermatogonial proliferation in a Tp53 knockout rat. Andrology.

[26] Blanco-Rodríguez J (2009). gammaH2AX marks the main events of the spermatogenic process. Microsc. Res. Tech..

[27] Josefa B-R (2012). Programmed phosphorylation of histone H2AX precedes a phase of DNA double-strand break-independent synapsis in mouse meiosis. Reproduction.

[28] Komeya M (2019). In vitro spermatogenesis in two-dimensionally spread mouse testis tissues. Reprod. Med. Biol..

[29] Lindsey JR, Baker HJ, Lindsey JR, Weisbroth SH (1979). Chapter 1—historical foundations. The Laboratory Rat.

[30] Hashway SA, Wilding LA, Suckow MA, Hankenson FC, Wilson RP, Foley PL (2020). Chapter 3—translational potential of rats in research. The Laboratory Rat.

[31] Miyake K, Yamamoto M, Mitsuya H (1986). Pharmacological and histological evidence for adrenergic innervation of the myoid cells in the rat seminiferous tubule. Tohoku J. Exp. Med..

[32] Hakamata Y (2001). Green fluorescent protein-transgenic rat: a tool for organ transplantation research. Biochem. Biophys. Res. Commun..

[33] Russell LD, Ettlin RA, Hikim APS, Clegg ED (1993). Histological and Histopathological Evaluation of the Testis. Int. J. Androl..

[34] Champy CH (1920). De la méthode de culture des tissus. VI. Le testicule. Arch. Zool. Exp. Gen..

[35] Steinberger A, Steinberger E (1970). Tissue culture of male mammalian gonads. vitro.

[36] Gohbara A (2010). In vitro murine spermatogenesis in an organ culture system. Biol. Reprod..

[37] Buehr M (2008). Capture of authentic embryonic stem cells from rat blastocysts. Cell.

[38] Li P (2008). Germline competent embryonic stem cells derived from rat blastocysts. Cell.

[39] Li W (2009). Generation of rat and human induced pluripotent stem cells by combining genetic reprogramming and chemical inhibitors. Cell Stem Cell.

[40] Liao J (2009). Generation of induced pluripotent stem cell lines from adult rat cells. Cell Stem Cell.

[41] Ueda S (2008). Establishment of rat embryonic stem cells and making of chimera rats. PLoS ONE.

[42] Zhou Q (2003). Generation of fertile cloned rats by regulating oocyte activation. Science.

[43] Hirabayashi M (2002). Offspring derived from intracytoplasmic injection of transgenic rat sperm. Transgenic Res..

[44] Hirabayashi M, Kato M, Aoto T, Ueda M, Hochi S (2002). Rescue of infertile transgenic rat lines by intracytoplasmic injection of cryopreserved round spermatids. Mol. Reprod. Dev.: Inc. Gamete Res..

[45] Johnson F, Sinclair H (1979). The antioxidant vitamins. Crit. Rev. Food Sci. Nutr..

[46] Bensoussan K, Morales CR, Hermo L (1998). Vitamin E deficiency causes incomplete spermatogenesis and affects the structural differentiation of epithelial cells of the epididymis in the rat. J. Androl..

[47] Steinberger A (1967). Factors affecting spermatogenesis in organ cultures of mammalian testes. J. Reprod. Fertil. Suppl..

[48] Quinn P, Harlow GM (1978). The effect of oxygen on the development of preimplantation mouse embryos in vitro. J. Exp. Zool..

[49] Karagenc L, Sertkaya Z, Ciray N, Ulug U, Bahçeci M (2004). Impact of oxygen concentration on embryonic development of mouse zygotes. Reprod. BioMed. Online.

[50] Gholami K, Vermeulen M, Del Vento F, de Michele F, Giudice MG, Wyns C (2020). The air-liquid interface culture of the mechanically isolated seminiferous tubules embedded in agarose or alginate improves in vitro spermatogenesis at the expense of attenuating their integrity. Vitro Cell. Dev. Biol. Anim..

[51] Lysiak JJ, Nguyen QAT, Turner TT (2000). Fluctuations in rat testicular interstitial oxygen tensions are linked to testicular vasomotion: persistence after repair of torsion1. Biol. Reprod..

[52] Sato T (2011). In vitro production of fertile sperm from murine spermatogonial stem cell lines. Nat. Commun..

[53] Kojima K (2018). Neonatal testis growth recreated in vitro by two-dimensional organ spreading. Biotechnol. Bioeng..

[54] Duffy DC, McDonald JC, Schueller OJ, Whitesides GM (1998). Rapid prototyping of microfluidic systems in poly(dimethylsiloxane). Anal. Chem..

[55] Fujii T (2002). PDMS-based microfluidic devices for biomedical applications. Microelectron. Eng..

[56] Sato T, Katagiri K, Kubota Y, Ogawa T (2013). In vitro sperm production from mouse spermatogonial stem cell lines using an organ culture method. Nat. Protoc..

