# Supplementary material for: Rat in vitro spermatogenesis promoted by chemical supplementations and oxygen-tension control
Source: Sci Rep. 2021 Feb 10;11:3458. doi: 10.1038/s41598-021-82792-2 (PMC7875995; doi:10.1038/s41598-021-82792-2)
Supplement: Supplementary file 1 — Supplementary Information 1. [file 41598_2021_82792_MOESM1_ESM.pdf]

Supplementary information

Rat *in vitro* spermatogenesis promoted by chemical supplementations and oxygen-tension control

Takafumi Matsumura[1], Takuya Sato[1], Takeru Abe[1], Hiroyuki Sanjo[2], Kumiko Katagiri[1], Hiroshi Kimura[3], Teruo Fujii[4], Hiromitsu Tanaka[5], Masumi Hirabayashi[6], Takehiko Ogawa[1, 2 *]

[1] Laboratory of Biopharmaceutical and Regenerative Sciences, Institute of Molecular Medicine and Life Science, Yokohama City University Association of Medical Science, Yokohama, Kanagawa, Japan.
[2] Department of Urology, Yokohama City University School of Medicine, Yokohama, Kanagawa, Japan.
[3] Department of Mechanical Engineering, Tokai University, Hiratsuka, Kanagawa, Japan
[4] Institute of Industrial Science, University of Tokyo, Bunkyo, Tokyo, Japan
[5] Faculty of Pharmaceutical Sciences, Nagasaki International University, Sasebo, Nagasaki, Japan.
[6] Center for Genetic Analysis of Behavior, National Institute for Physiological Sciences, Okazaki, Aichi, Japan.

*To whom correspondence may be addressed. Email: ogawa@yokohamacu.ac.jp

Supplementary Figure

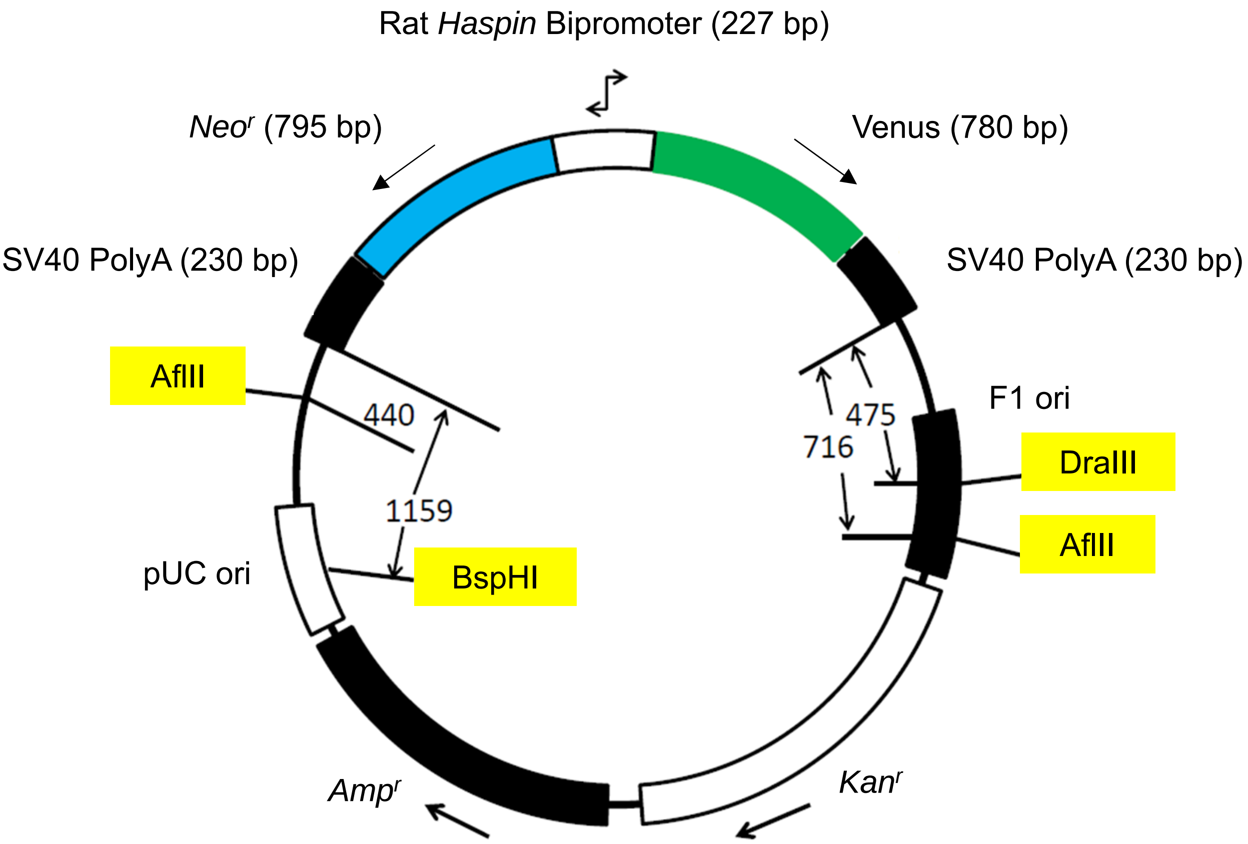

Supplementary Figure 1. The plasmid used to generate *Haspin*-Venus Tg rat. Venus and neomycin resistance gene (*Neo^r*) are expressed under the *Haspin* bidirectional promoter. Arrows indicate the direction of translation. The yellow boxes indicate the restriction enzyme recognition sites.

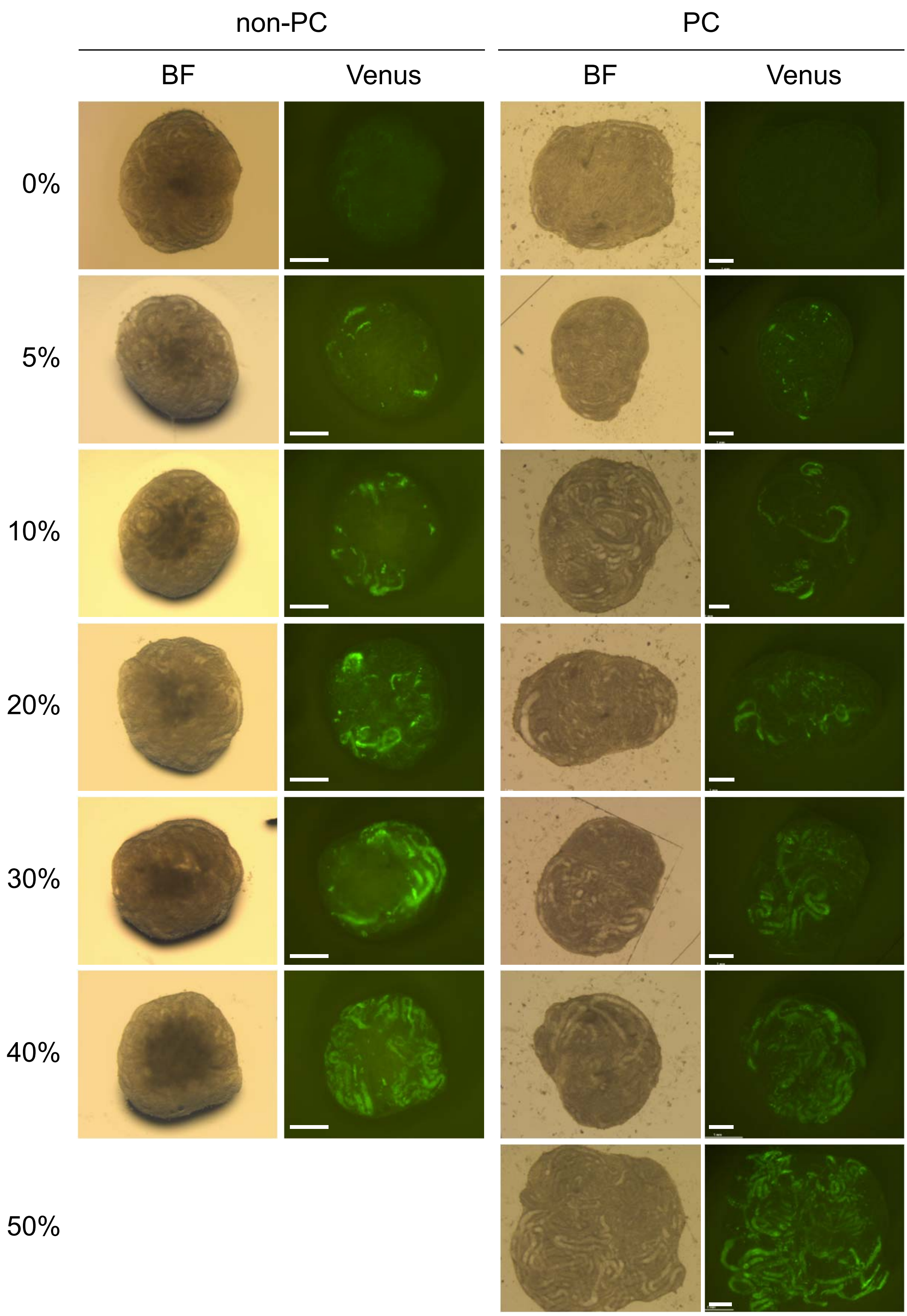

Supplementary Figure 2. Grading of cVenus expression. The extent of central Venus (cVenus) expression in the cultured testicular tissue was classified into 7 grades (0%, 5%, 10%, 20%, 30%, 40%, 50%) according to the percent area of cVenus expression in the whole region of each tissue fragment. In non-PC group, 50% cVenus expression was not observed in this study. Scale bar = 500 µm.

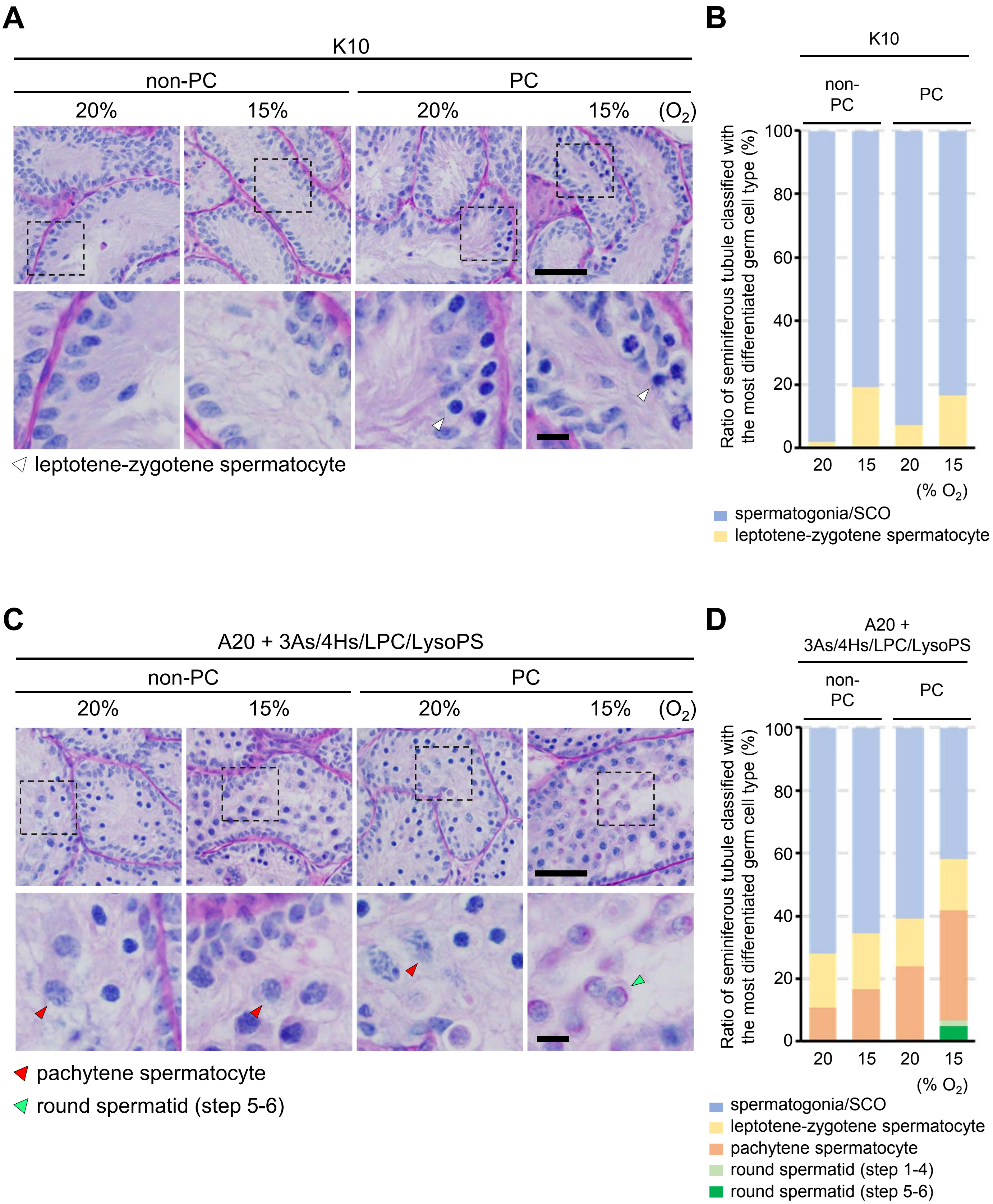
A
K10
non-PC
PC
20%
15%
20%
15%
(O2)
leptotene-zygotene spermatocyte
B
K10
non-PC
PC
Ratio of seminiferous tubule classified with the most differentiated germ cell type (%)
100
80
60
40
20
0
20
15
20
15
(% O2)
spermatogonia/SCO
leptotene-zygotene spermatocyte
C
A20 + 3As/4Hs/LPC/LysoPS
non-PC
PC
20%
15%
20%
15%
(O2)
pachytene spermatocyte
round spermatid (step 5-6)
D
A20 + 3As/4Hs/LPC/LysoPS
non-PC
PC
Ratio of seminiferous tubule classified with the most differentiated germ cell type (%)
100
80
60
40
20
0
20
15
20
15
(% O2)
spermatogonia/SCO
leptotene-zygotene spermatocyte
pachytene spermatocyte
round spermatid (step 1-4)
round spermatid (step 5-6)

Supplementary Figure 3. *In vitro* spermatogenesis with wild-type SD rats.

(**A**) Histological pictures of SD rat testis tissues cultured for 6 weeks using αMEM + 10% KSR (K10) medium. Dashed line rectangle areas in the top panels were enlarged in the bottom panels. Scale bar; 50 µm (top panels), 10 µm (bottom panels). White arrowheads indicate leptotene-zygotene spermatocyte. (**B**) Ratio of seminiferous tubules classified with the most differentiated germ cell type, with culture medium of K10. (**C**) Histological pictures of SD rat testis tissues cultured for 6 weeks using medium, αMEM + AlbuMAX (20 mg/mL), 3As, 4Hs, LPC, and LysoPS. Red and green arrowheads indicate pachytene spermatocyte and step 5 – 6 round spermatids, respectively. Dashed line rectangle areas in the top panels were enlarged in the bottom panels. Scale bar; 50 µm (top panels), 10 µm (bottom panels). (**D**) Ratio of seminiferous tubules classified with the most differentiated germ cell type, with culture medium of A20 + 3As/4Hs/LPC/LysoPS. Specimens were stained with hematoxylin and periodic acid Schiff (PAS).

Supplementary Tables

Ingredients of culture medium		
AlbuMAX I	10-40	mg/mL
Testosterone	1	μM
3,3′,5-Triiodo-L-thyronine sodium	2	ng/mL
LH	1	ng/mL
FSH	1	ng/mL
L-Ascorbic acid 2 glucoside	1	mM
DL-α-Tocopherol acetate	1	mM
L-Glutathione reduced	1	mM
L-α-Lysophosphatidylcholine	100	μg/mL
Lysophosphatidylserine	1	μM

These ingredients were added to the basal medium of α-MEM.

Supplementary Table 1. Ingredients in the culture medium used for rat *in vitro* spermatogenesis. The final concentration of each ingredient was on the right column.

Medium	PC covering	O_2 (%)	Culture period (weeks)	Number of tissues histologically examined	Age of donor rats, postnatal day (# tissue)	Tissues containing round spermatid, step 1-6 (%)	Tissues containing round spermatid, step 5-6 (%)
A20 + 3As/4Hs	-	20	6	6	P7	4 (66.7)	1 (16.7)
	+	20	6	13	P5 (1), P7 (12)	9 (69.2)	5 (38.5)
	-	15	6	12	P7	9 (75.0)	7 (58.3)
	+	15	6	5	P7	5 (100)	1 (20.0)
	-	10	6	9	P7	7 (77.8)	5 (55.6)
	+	10	6	5	P7	1 (20.0)	0 (0)
	+	20	10 - 11	5	P7 (3), P9 (2)	1 (20.0)	1 (20.0)
A20 + 3As/4Hs/LPC/LysoPS	+	20	6	8	P4 (2), P5 (1), P7 (5)	5 (62.5)	2 (25.0)
	+	20	10 - 11	5	P7 (3), P9 (2)	4 (80.0)	3 (60.0)

Supplementary Table 2. Summary of culture results histologically evaluated. The cultured testis fragments were evaluated histologically, with hematoxylin and PAS staining, to identify round spermatids. Step 1-6 includes all spermatids found. Step 5-6 spermatids had clearly visible cap-shaped acrosome stained by PAS.

Supplementary Table 3 is presented separately in another file.

Supplementary Table 3. Original data for each cultured tissue used in the chart in the figure. (A) Data for each cultured tissue used in Figure 2F, including litter ID, rat ID, rat age, medium, PC usage, oxygen concentration, and cVenus expression grade are shown. (B) Data for each cultured tissue used in Figure 3A, including litter ID, rat ID, rat age, medium, PC usage, oxygen concentration, and cVenus expression grade in each week are shown. (C) Data for each cultured tissue used in Figure 4B, including litter ID, rat ID, rat age, medium, PC usage, oxygen concentration, and cVenus expression grade are shown. (D) Data for each cultured tissue used in Figure 4E, including litter ID, rat ID, rat age, medium, PC usage, oxygen concentration, and histological evaluation are shown. (E) Data for each cultured tissue used in Figure 5B, including litter ID, rat ID, rat age, medium, PC usage, oxygen concentration, and cVenus expression grade in each week are shown. (F) Data for each cultured tissue used in Figure 5E, including litter ID, rat ID, rat age, medium, PC usage, oxygen concentration, and histological evaluation are shown. (G) Data for each cultured tissue used in Supplementary Figure 3B and D, including litter ID, rat ID, rat age, medium, PC usage, oxygen concentration, and histological evaluation are shown.
